# Artificial Intelligence-Driven Personalization in Breast Cancer Screening: From Population Models to Individualized Protocols

**DOI:** 10.3390/cancers17172901

**Published:** 2025-09-04

**Authors:** Filippo Pesapane, Luca Nicosia, Lucrezia D’Amelio, Giulia Quercioli, Mariassunta Roberta Pannarale, Francesca Priolo, Irene Marinucci, Maria Giorgia Farina, Silvia Penco, Valeria Dominelli, Anna Rotili, Lorenza Meneghetti, Anna Carla Bozzini, Sonia Santicchia, Enrico Cassano

**Affiliations:** 1 Breast Imaging Division, IEO European Institute of Oncology IRCCS, 20141 Milan, Italy; luca.nicosia@ieo.it (L.N.); francesca.priolo@ieo.it (F.P.); irene.marinucci@ieo.it (I.M.); mariagiorgia.farina@ieo.it (M.G.F.); silvia.penco@ieo.it (S.P.); valeria.dominelli@ieo.it (V.D.); anna.rotili@ieo.it (A.R.); lorenza.meneghetti@ieo.it (L.M.); anna.bozzini@ieo.it (A.C.B.); enrico.cassano@ieo.it (E.C.); 2 Diagnostic and Interventional Radiology Department, SC Radiologia, IRCCS Cà Granda Fondazione Ospedale Maggiore Policlinico, 20122 Milan, Italy; damelio.lucrezia@gmail.com (L.D.); sonia.santicchia@policlinico.mi.it (S.S.); 3 Department of Medical Surgical Sciences and Translational Medicine, Sapienza University of Rome, Radiology Unit, Sant’Andrea University Hospital, 00189 Rome, Italy; 4 Postgraduate School in Radiodiagnostics, Università degli Studi di Milano, 20122 Milan, Italy; giulia.quercioli@unimi.it (G.Q.); mariassunta.pannarale@unimi.it (M.R.P.)

**Keywords:** artificial intelligence, breast cancer screening, risk stratification, personalized medicine, mammography, clinical decision, support systems

## Abstract

Conventional breast cancer screening programs are predominantly age-based, applying uniform intervals and modalities across broad populations. While this model has reduced mortality, it entails harms—including overdiagnosis, false positives, and missed interval cancers—prompting interest in risk-stratified approaches. In recent years, artificial intelligence (AI) has emerged as a critical enabler of this paradigm shift. This narrative review examines how AI-driven tools are advancing breast cancer screening toward personalization, with a focus on mammographic risk models, multimodal risk prediction, and AI-enabled clinical decision support. We reviewed studies published from 2015 to 2025, prioritizing large cohorts, randomized trials, and prospective validations. AI-based mammographic risk models generally improve discrimination versus classical models and are being externally validated; however, evidence remains heterogeneous across subtypes and populations. Emerging multimodal models integrate genetics, clinical data, and imaging; AI is also being evaluated for triage and personalized intervals within clinical workflows. Barriers remain—explainability, regulatory validation, and equity. Widespread adoption will depend on prospective clinical benefit, regulatory alignment, and careful integration. Overall, AI-based mammographic risk models generally improve discrimination versus classical models and are being externally validated; however, evidence remains heterogeneous across molecular subtypes, with signals strongest for ER-positive disease and limited data for fast-growing and interval cancers. Prospective trials demonstrating outcome benefit and safe interval modification are still pending. Accordingly, adoption should proceed with safeguards, equity monitoring, and clear separation between risk prediction, lesion detection, triage, and decision-support roles

## Simple Summary

Breast cancer screening reduces mortality, yet uniform, age-based schedules still cause false positives, overdiagnosis, and missed interval cancers. This review examines how artificial intelligence could enable risk-stratified screening and distinguishes four functions: risk prediction, lesion detection, workflow triage, and clinical decision support. We summarize current evidence, highlight uncertainties for aggressive subtypes and dense breasts, and propose safeguards for responsible use, including prospective outcome trials, local calibration, monitoring of interval cancers by subtype, and equity audits. We also outline regulatory and operational considerations. Our aim is to guide researchers toward robust study designs and transparent reporting that advance safe, clinically meaningful personalization.

## 1. Introduction

Breast cancer screening has historically followed a one-size-fits-all approach, with eligibility and frequency determined primarily by age. While age-based population screening programs (e.g., inviting all women 50–69 for biennial mammography) have reduced breast cancer mortality, they also incur substantial collateral harms [1,2,3]. Key drawbacks of the traditional paradigm—notably overdiagnosis of indolent tumors, high false-positive recall rates, and uneven risk-benefit tradeoffs—have prompted calls to refine screening on an individualized basis. In principle, tailoring screening intensity and modality to each woman’s risk profile could improve the benefit-to-harm ratio by detecting aggressive cancers earlier while sparing low-risk women from unnecessary procedures [1,3,4].

Realizing this vision, however, requires accurate tools for risk stratification and decision support at the individual level. Artificial intelligence (AI) has emerged as a catalytic technology in this domain [5]. This narrative review critically examines the shortcomings of the current breast cancer screening approach and explores how AI-driven risk models and decision tools are transforming it toward personalization, also addressing challenges such as model explainability, clinical workflow integration, regulatory validation, and ethical implications. The four distinct AI functions in breast screening are summarized in Table 1.

We searched MEDLINE/PubMed, Embase, Scopus, Web of Science Core Collection, and the Cochrane Library for English-language studies from January 2015 to August 2025, using combinations of: “AI OR deep learning OR machine learning” AND “breast cancer screening” AND (“risk stratification” OR “risk model” OR “triage” OR “decision support” OR “personalized screening”). We also screened ClinicalTrials.gov and the EU Clinical Trials Register for ongoing or completed studies, and hand-searched reference lists of key reviews and trials. We prioritized prospective/randomized evidence and large, externally validated cohorts; modeling and high-quality retrospective studies were included when informative.

## 2. From Traditional to Personalized Breast Cancer Screening

The current conventional breast screening programs have some limitations, essentially due to the fact that they target broad age cohorts with fixed schedules, an approach that overlooks individual variability in risk and tumor behavior. Overdiagnosis—the detection of cancers (often low-grade or ductal carcinoma in situ) that would not have become life-threatening—is a serious harm of population mammography screening [3,6]. Estimates of overdiagnosis vary widely, but even conservative analyses confirm that a non-trivial fraction of screen-detected breast cancers represent overdiagnosis [6]. Accordingly, overdiagnosis may lead to overtreatment (surgery, radiotherapy, etc.) of lesions that would never have threatened the patient’s life [6]. This not only causes avoidable morbidity and anxiety for women but also dilutes the net mortality benefit of screening [3].

False-positive results are another major drawback: mammography’s limited specificity means that many women are recalled for additional imaging or biopsies that ultimately prove benign. The cumulative risk of a false-positive screening mammogram is substantial: contemporary cohort data from the U.S. Breast Cancer Surveillance Consortium showed that ~50–60% of women undergoing ten years of annual 2D mammography will experience at least one false-positive recall [7]. A false-positive result often triggers short-term anxiety and invasive follow-up (e.g., an unnecessary needle biopsy in ~11% of women over ten years of annual screens) [7]. These inefficiencies underscore that current screening expends substantial effort on women who do not have cancer, while still missing some cancers [3].

Indeed, interval cancers and advanced-stage diagnoses persist under age-based screening. Even in optimized programs, around 20–25% of breast cancers present between scheduled screens (interval cancers) or are diagnosed at stage II or higher despite regular screening [8]. This reflects the limitation of time-fixed screening intervals that may be too infrequent for higher-risk women, as well as the variable tumor growth rates that “one-speed” screening cannot accommodate.

Collectively, these issues—overdiagnosis, false positives, missed interval cancers, and inefficient allocation of screening resources—have prompted a re-examination of the age-based paradigm. There is a growing consensus that risk-stratified screening could better balance benefits and harms [6]. Women at higher risk (due to factors like family history, genetic predisposition, dense breasts, etc.) might benefit from earlier or more intensive screening with sensitive modalities, whereas low-risk women could be screened less often or with different methods, reducing unnecessary interventions [4,5,9]. Modeling studies and patient preference analyses suggest many women will accept a tailored approach if it maintains or improves cancer detection while reducing false positives and overdiagnosis [4,5,6,9]. However, implementing such personalization in practice is challenging—it requires accurate risk assessment tools and evidence that tailoring does not compromise outcomes. Traditional risk models (e.g., Gail, Tyrer–Cuzick) have been developed to estimate individual breast cancer risk, but these rely on limited risk factors and have shown only modest discriminatory accuracy (often with AUC < 0.65–0.70) [5,10].

The concept of personalized (risk-based) breast cancer screening has moved from theoretical appeals to real-world trials in the past decade: the ENVISION consensus in 2019 identified risk-stratified early detection as a priority, calling for “evidence-based personalized interventions that might improve benefits and reduce harms of existing screening programmes” [11]. In such a model, women would follow individualized screening schedules (frequency and modality) based on their calculated risk, rather than uniform age criteria. Two large randomized trials are actively testing this paradigm: the WISDOM trial in the United States (US) [12] and the MyPeBS trial in Europe [10]. WISDOM (Women Informed to Screen Depending on Measures of Risk) is comparing an annual age-based regimen to a personalized schedule (which incorporates genetic, clinical, and breast density risk factors) in 100,000 women to assess whether risk-guided screening is as safe and effective as standard screening [12]. MyPeBS (My Personal Breast Screening) similarly randomizes women to risk-based vs. standard screening to evaluate cancer outcomes and harms [10,13]. These landmark studies will provide high-level evidence on the feasibility and impact of personalization.

In parallel, AI methods are rapidly advancing the accuracy of risk assessment, which is the linchpin of personalization [5]. Deep learning algorithms can mine high-dimensional data (such as every pixel of a mammogram) for subtle signatures of risk that elude human observers and simplistic models [14,15]. Notably, a woman’s mammographic images contain rich latent information about future cancer risk beyond the obvious findings like breast density: AI algorithms can “learn” these latent patterns from large training sets of images with known outcomes [16]. The past few years have seen the development of powerful AI-based risk models that substantially outperform classical risk models on discrimination and reclassification of risk [17]. Moreover, some AI models combine multiple data modalities—imaging, clinical risk factors, genomics—to yield a more holistic risk prediction [16]: this is the juncture at which AI promises to make a decisive impact: by leveraging large datasets of imaging and clinical variables, AI-based models are dramatically improving risk prediction and enabling dynamic, personalized screening strategies [5], as summarized in Table 2.

## 3. AI in Mammographic Risk Prediction

AI mammographic risk models leverage image-derived phenotypes to stratify future risk and generally outperform classical clinical models on discrimination. However, reported performance is not uniform across endpoints or subgroups, and evidence on fast-growing and interval cancers is still maturing. These considerations are crucial before using AI scores to extend intervals in low-risk women. Some features on mammograms, such as breast density, architectural distortions, or tissue texture, correlate with a woman’s future cancer risk [4,5,10,18,19,20]. While traditional approaches incorporated one such feature—percent breast density—into risk models, modestly improving their accuracy, an AI tool can take this much further by processing the entire mammogram to identify complex imaging phenotypes of risk [5,10]. These models essentially act as an “algorithmic second read” of a normal mammogram, not to find an existing cancer, but to predict the likelihood of a cancer emerging in the next few years [16,19]. Yala et al. [19] developed a deep learning model using thousands of mammograms to predict 5-year cancer risk; by 2021, this evolved into the Mirai model, which was designed to produce consistent risk estimates across time points and imaging equipment. Mirai achieved impressive accuracy: in independent test sets from the US, Sweden, and Taiwan, it attained concordance indices of 0.76–0.81 for 5-year risk prediction [16,19,21]. This substantially exceeded the performance of the Tyrer–Cuzick (IBIS) risk model, which had AUCs in the mid-0.60s on the same cohorts [5]. In practical terms, Mirai was able to identify a much larger fraction of women who would develop cancer within 5 years as “high-risk.” Yala et al. [19] reported that on the Massachusetts General Hospital dataset, the top 10% of women by Mirai risk captured ~41.5% of those who actually developed cancer in 5 years, compared to only ~23% captured by Tyrer–Cuzick. In other words, AI nearly doubled the yield of high-risk stratification, which could translate to targeting preventive measures (like MRI or chemoprevention) more effectively. These findings highlight how AI can unveil discriminatory image features (e.g., subtle tissue heterogeneity or vascular patterns) that traditional risk factors fail to account for.

Other teams have developed similar mammogram-based risk models with strong results. A deep learning model by Damiani et al. (trained and tested on UK screening data) reached an AUC of 0.68 for 3-year cancer risk prediction from a negative mammogram [22]. Notably, its performance was consistent for cancers detected at routine screening vs. interval cancers (AUC ~0.67–0.69 for both), and it was better at predicting more aggressive cancers—the AUC for later-stage (Stage II+) cancer was 0.72 [22]. The authors concluded this AI model was a “strong predictor” of risk 3–6 years out, and it was well calibrated across subgroups.

Eriksson et al. [10] validated an AI-derived risk model (the ProFound AI Risk model by iCAD) across four countries’ screening cohorts. In a nested case–control study including >8500 women, the image-based AI risk score achieved an adjusted AUC of ~0.72 for 2-year risk. Importantly, this performance generalized across diverse populations (Sweden, Spain, Italy, Germany) without loss of accuracy. Using established risk thresholds, about 6% of women were classified as high-risk by the AI model; these women had a 6.7-fold higher likelihood of developing cancer before the next screen compared to those at general risk. Put differently, the model could pinpoint a small subset of women (6%) who accounted for roughly 30% of all stage II+ cancers occurring within two years despite an initial negative mammogram. Such women might be candidates for supplemental screening immediately or at a shorter interval, illustrating AI’s potential to flag those “missed” by standard screening for earlier [10]. Notably, the AI’s risk stratification remained effective even in women with dense breasts, a group in whom traditional mammography is less sensitive.

Overall, these studies demonstrate that AI mammographic risk models consistently achieve AUCs in the 0.70–0.80 range, significantly outperforming older clinical risk models (most of which languish below 0.65–0.70). The improvements are not just statistically significant but clinically meaningful—enabling identification of high-risk cohorts with much greater confidence. In fact, Eriksson et al. [10] noted that AI-based risk assessment now represents a “mature technology designed for risk-stratified screening” and could justify pragmatic trials integrating risk scoring into screening programs. Some AI risk models have even entered clinical use: ProFound AI Risk, for instance, is FDA-cleared and CE-marked, allowing radiologists to obtain an instantaneous 1- to 2-year risk estimate whenever they interpret a screening mammogram [23,24]. This is a paradigm shift—risk assessment moving from a separate clinic-based calculation to an automated output alongside the imaging read.

Despite this progress, challenges remain. Most AI risk models to date have been developed and validated retrospectively; prospective trials are needed to show that acting on AI risk predictions improves outcomes. External validations, while promising, are still relatively few [16,19,21,25]. Moreover, these models function largely as “black boxes,” raising questions about how to explain a high-risk score to a patient or clinician [15,26]. Nonetheless, the field is moving fast: the convergence of large longitudinal image datasets, improved algorithms, and computational power has made mammographic risk prediction one of the first real AI success stories in breast screening [19].

Available studies indicate heterogeneity by tumor phenotype [10,16,18,22,24]. Image-based short-term risk has shown enrichment of later-stage/aggressive phenotypes in the high-risk stratum when combined with genetics and lifestyle factors, while signals are strongest for ER-positive disease and less certain for triple-negative breast cancers (TNBCs). Several works report similar discrimination for screen-detected and interval cancers over 3-year horizons, but precise estimates for ER-negative/TNBC remain limited and warrant prospective evaluation before interval extension in younger, dense-breasted populations [15,18,22,23,24]. Consequently, any policy using AI to reduce screening intensity should cap interval extension pending prospective safety data, track interval cancers by subtype and density, and include pre-specified stopping rules if interval cancer rates rise in low-risk strata, as summarized in Table 3.

## 4. Multimodal Data Integration for Individualized Risk

While mammograms alone carry a significant predictive signal, breast cancer risk is multifactorial. An individual’s risk is influenced by genetic factors (e.g., BRCA or polygenic risk scores), reproductive and hormonal history, lifestyle (obesity, alcohol), family history, breast density, and more [27,28]. Multimodal AI risk models aim to integrate these diverse inputs—imaging plus traditional risk factors and even genomic data—to provide a more comprehensive risk assessment [29,30,31,32,33,34,35,36].

One approach adds clinical risk factors and breast density into the AI modeling: Eriksson et al. [23] built an “extended” risk model where an image-based risk score (derived from mammographic features) was combined with lifestyle factors and a polygenic risk score of 313 SNPs. In a cohort of ~70,000 Swedish women, the purely image-based model achieved AUC 0.73 for 2-year risk, which improved to 0.74 when adding lifestyle factors and to 0.77 when adding genetic risk. High-risk women identified by this combined model had an eightfold higher risk of cancer than those at low risk. Moreover, the high-risk group was enriched for cancers with aggressive features—they were more likely to develop stage II tumors ≥20 mm and less likely to have indolent features like small, ER-positive tumors. This suggests multimodal models cannot only predict risk quantity but also risk quality (i.e., the likelihood of a dangerous cancer). Such women clearly stand to benefit from intensified screening (or prevention). Indeed, in Sweden, a 2-year risk above a certain threshold (e.g., ≥ 2.5%) qualifies a woman for supplemental MRI by law [37]. In the Eriksson study, roughly 12% of women exceeded that threshold according to the AI+genetics model, versus fewer by older models [23], indicating more candidates for early intervention would be identified.

Beyond mammography and clinical data, researchers are exploring AI integration of other imaging modalities, like breast MRI-based, particularly for high-risk populations for which breast MRI is particularly indicated [37].

Multimodal AI is also extending to pathology and genomics in the broader breast cancer care continuum (predicting prognosis or therapy response) [30,32,33,34,35,36,38,39], but in the screening context, the main focus has been on combining imaging with risk factors [40]. Multimodal AI approaches hold the promise of truly individualized risk prediction—the algorithmic equivalent of a comprehensive breast cancer risk consult. By assimilating all relevant data (a recent mammogram’s AI score, the patient’s reproductive history and genetics, etc.), such models could categorize women into nuanced risk tiers that inform not only if and when to screen, but also how. A low-risk woman might safely undergo less frequent digital mammography, whereas a high-risk woman (say with high AI image score + strong family history) might be triaged to annual MRI or supplemental ultrasound [5].

## 5. AI-Enabled Triage and Detection in Screening Workflow

Personalization in screening is not only about risk prediction; it also involves adapting the screening process itself to individual needs and leveraging AI to improve efficiency. A key application of AI in breast screening is as a “third eye” or independent reader of mammograms to aid radiologists. Numerous AI algorithms have been developed to detect breast cancer on mammograms, some achieving radiologist-level sensitivity in retrospective studies [14,41,42,43,44,45,46]. The conventional deployment of such AI has been to assist with lesion detection and reduce human reading errors. But in the context of screening personalization, AI detection can be harnessed for triage automation—deciding which exams or patients warrant more urgent attention or additional work-up [43,47,48].

One strategy is using AI as an independent first reader in screening, effectively to triage normal from suspicious exams. In regions like Europe, where double-reading of mammograms is standard, there is intense interest in whether one radiologist could be safely replaced by AI, thereby addressing workforce shortages and maintaining quality [3,49,50,51,52]. In a landmark Swedish trial (ScreenTrustCAD) [44], 55,000 women were screened with an AI system inserted into the reading workflow were screened with an AI system inserted into the reading workflow. Every mammogram was read in parallel by: (a) two radiologists (standard), (b) one radiologist plus AI, (c) AI alone, and (d) two radiologists plus AI, to compare cancer detection and false positives under each scenario. The findings were highly encouraging: One radiologist + AI was non-inferior to two radiologists for cancer detection, actually detecting 4% more cancers (261 vs. 250 cases, a relative increase of 1.04) with a similar recall rate. AI alone as a single reader also performed comparably to two radiologists (98% of the cancer detection rate). There was no significant increase in false positives with AI in the mix, and the combination of two radiologists + AI detected ~8% more cancers than two without AI. The authors concluded that replacing one reader with AI maintained screening accuracy while modestly improving detection, suggesting “AI in the study setting has potential for controlled implementation” in population screening. Another trial, MASAI [43], conducted in Europe (Sweden and elsewhere), similarly reported that AI-supported reading can safely reduce radiologist workload without missing cancers. These prospective studies move the evidence base beyond retrospective enrichment studies, confirming in real practice that AI can indeed shoulder part of the screening burden.

The implications for personalization are notable. By automating triage of obvious negatives, AI could allow radiologists to focus on exams that are borderline or high-risk. For instance, a workflow might let AI outright “clear” a subset of low-risk, normal mammograms (with periodic audit for safety), while escalating those with high AI suspicion scores for double-reading or expedited assessment. This kind of stratified workflow would personalize the reading intensity to the case at hand—essentially adaptive reading. Indeed, some authors envision AI-driven risk-based reading intervals: if an exam is scored as very low risk by AI, the patient could be invited back in two years instead of one, whereas a high-score exam might prompt an immediate work-up or 6-month follow-up even if the radiologist did not see a lesion [53]. Early health-economic analyses indicate that using AI in this triage capacity can be cost-effective, by preventing cancer cases through earlier detection and reducing unnecessary recalls [17]. Beyond reading mammograms, AI is being applied to automate other triage decisions in screening. One example is automating referrals to supplemental imaging. Women with extremely dense breasts have lower mammographic sensitivity and often benefit from supplemental ultrasound or MRI. AI could identify which dense-breasted women actually have sufficient risk to justify MRI—a task currently guided crudely by density alone or simple models [5]. Studies used AI scores to decide which women with dense breasts got a supplemental imaging (breast ultrasound or breast MRI) showing that result was a higher cancer detection rate compared to mammography alone, showing promise for AI-tailored modality selection: their combined risk model could flag a subset of women with ~8-fold risk who indeed had more interval cancers; these women might be funneled to receive MRI or ultrasound immediately instead of waiting two years [23,54].

AI-based triage also extends to workflow optimization. Some screening programs are exploring AI to prioritize the reading queue—for instance, routing mammograms with high AI suspicion scores to be read first or by more experienced radiologists, ensuring prompt action on likely cancers, while letting low-score exams wait a bit longer. This time-sensitive triage could shorten the time to diagnosis for aggressive cancers. Moreover, AI might reduce the need for consensus recall conferences by providing a concurrent second opinion: if one radiologist sees an abnormality and AI strongly agrees, the recall decision could be more straightforward, whereas discordant cases might undergo further review.

Notably, these implementations require careful clinical integration, radiologists (and patients) trust in AI, and regulatory approval for such use are all essential, but the evidence to date indicates that AI can maintain or even enhance cancer detection when used as an adjunct or partial substitute for human readers [25,55,56,57,58].

Notably, this section concerns lesion detection and triage on the current exam and does not address long-term risk prediction.

Ultimately, AI-driven triage serves the larger goal of personalized screening by aligning resources with risk: higher risk or suspicious cases get more attention and possibly different modalities, while low-risk cases avoid excess interventions.

## 6. AI-Driven Clinical Decision Support for Personalized Protocols

Personalizing breast screening requires not only risk estimation and detection, but also translating those insights into concrete clinical decisions: Here, we consider decision support, i.e., converting risk/detection outputs into schedules and modality choices. The main questions are: Who should start screening early? Who can safely defer or extend the interval? What modality is best for whom? These decisions are complex and multifactorial, which is where AI-powered clinical decision support (CDS) systems come into play. A CDS could synthesize a patient’s risk information (from AI models and other data) and recommend a tailored screening plan for the clinician and patient to consider.

Brentnall et al. [53] used linear programming on an AI risk score distribution to define risk-group-specific screening intervals that would minimize advanced cancers under a fixed resource budget Their model, applied to the UK screening context, suggested a three-tier protocol: the top ~4% of women by AI risk get annual mammograms, the middle ~64% continue with the standard 3-year interval, and the bottom ~32% extend to every 4 years. This approach kept overall screening frequency the same on average but was expected to reduce advanced cancer incidence by an estimated 18 per 1000 cases compared to uniform triennial screening. While such simulations need real-world validation, they illustrate how AI risk stratification can feed into protocol optimization—effectively turning risk data into actionable screening schedules.

Another area of decision support is modality selection. AI can help decide if a given woman should receive adjunct screening with ultrasound or MRI: for example, an AI model might predict that a woman’s risk of an “interval” cancer (one that mammography would miss) is high; the CDS could then recommend supplemental MRI for her, as suggested by Eriksson et al. [23]. Studies and trials that provided MRI to women with extremely dense breasts showed a significant increase in cancer detection [37,59,60,61]. An AI-augmented approach could refine such strategies by targeting MRI to dense-breasted women who also have high AI-predicted risk, rather than all women with dense breasts.

Triage of follow-up intensity is another decision point: AI could assist in deciding whether a finding (or a patient’s composite risk profile) warrants immediate biopsy versus short-term follow-up imaging. Some AI systems output malignancy likelihood scores for detected lesions, which radiologists can use in their BI-RADS assessment. If an AI assigns an extremely low probability of cancer to a subtle finding, a radiologist might be more comfortable opting for a 6-month follow-up rather than an immediate invasive biopsy. Conversely, a high AI score might prompt a biopsy recommendation even if the human reader is on the fence. Early studies of AI in diagnostic mammography suggest it can reduce benign biopsies by identifying truly low-risk lesions (one study showed a 37% reduction in false-positive recalls with an AI diagnostic aid) [62].

Incorporating these AI lesion evaluations into screening recall decisions is a form of personalization: the decision is tailored not just to the image appearance, but to the AI’s learned experience from thousands of similar cases. Crucially, any AI-driven CDS for screening should be validated in clinical trials. The WISDOM trial’s personalized arm can be seen as a form of CDS where a risk model guides the screening schedule: it uses a risk algorithm (including genetic testing) to assign women to different starting ages and intervals, and will measure outcomes like stage II+ cancers [12,63]. Results from such trials will inform how safe and effective these AI-informed recommendations are.

Additionally, user-centered design is needed: how to present AI risk results to patients and clinicians in an understandable way, and how to incorporate patient preferences, since a decision aid might accompany the AI output to help women weigh the pros and cons of more intensive screening if they are high risk (e.g., acknowledging the higher false-positive risk of MRI vs. the higher cancer yield) [49,64,65].

At present, clinical guidelines have not fully caught up with AI developments—most still stratify screening only by very coarse factors (age, and sometimes a binary “high risk” category for known mutation carriers). But professional bodies are closely watching the evidence. It is conceivable that in the near future, guidelines will include recommendations like: “Women with an AI-predicted 5-year risk above a certain threshold should be offered annual screening with MRI, whereas those with very low AI risk could extend mammography interval to 3 years.” Before that happens, regulators and guideline panels will demand robust validation and possibly randomized trial evidence [58,66].

## 7. Model Explainability and Transparency

A critical challenge with AI models, especially deep learning, is the opacity of their decision-making [25,26].

In breast screening personalization, this raises practical and ethical concerns: how do we justify to a patient that an algorithm deemed her high-risk and recommends an MRI? How can clinicians trust an AI triage that skips someone’s mammogram review? The issue of explainability is paramount. Many AI risk models function as black boxes that take in pixel intensities and output a risk score without a clear human-interpretable rationale [67,68,69,70]. This lack of transparency can undermine clinician acceptance and patient trust [49,64,65,71]. For example, radiologists in qualitative studies have expressed wariness if an AI cannot provide a reason for its risk assessment beyond a numeric score [72].

In the context of screening, explainability is not just nice to have—it is tied to accountability: if an AI misses a cancer or mis-stratifies someone, understanding the failure mode is important for assigning responsibility and improving the model.

Researchers are increasingly exploring techniques for Explainable AI (XAI) in medical imaging. For mammography-based models, common approaches include saliency maps or heatmaps highlighting which regions of the image influenced the risk prediction [67,68,69,70]. For instance, an AI might subtly focus on areas of proliferative fibroglandular pattern; an explainability tool could visualize that those textures in the upper outer quadrant contributed heavily to the high-risk score. There is ongoing research into more advanced explainability methods—such as concept-based models that quantify known risk phenotypes (like “breast density heterogeneity score”) as intermediate outputs [73].

Another aspect of explainability is communicating risks to patients. If a woman is told, “your 5-year risk per our AI is 8%, placing you in the top decile,” she may reasonably ask why. Historically, risk models like Gail could be explained by their input (family history, etc.). With AI, one might say: “It’s partly your breast density and some imaging features that the algorithm found similar to other patients who developed cancer.”

From a regulatory and ethical perspective, calls have been made for a degree of algorithmic transparency. While disclosing the entire complex model weights is neither feasible nor useful to end-users, manufacturers are encouraged to provide information on a model’s training data and performance limits [74,75]. For example, knowing that an AI was trained predominantly on women aged 50–70 in Europe informs users that its risk estimates for a 40-year-old may be extrapolated. Explainability also links to fairness: if an AI consistently flags certain subgroups as high-risk due to biases in training, explainability methods might help uncover those spurious correlations [3,76,77,78].

In summary, several domains—summarized in Table 4—still need to be addressed, and it is crucial to integrate AI personalization tools into clinical practice responsibly. Without it, clinicians may be reluctant to act on AI recommendations, and patients may distrust the personalized protocols offered.

## 8. Clinical Integration and Workflow Implementation

Introducing AI-driven personalization into breast screening workflows is as much a human challenge as a technical one.

First, there is the issue of workflow fit. Radiology workflows are finely tuned for efficiency—adding an AI risk score or an AI reader means extra information that must be displayed, interpreted, and acted upon [15]. Ideally, AI outputs should be presented seamlessly within existing reporting systems (for example, directly on the PACS viewer or reporting software). If a radiologist has to log into a separate application to retrieve an AI risk report, it may not be used consistently.

Trials like ScreenTrustCAD integrated the AI such that radiologists received an AI decision (clear vs. recall suggestion) as part of their reading, which likely facilitated adoption [44]. In implementing personalized intervals, integration might mean the screening IT system automatically assigns the next appointment based on the AI risk category, which could be complex if some women go to 1-year and others to 3-year recalls in the same program.

Training and user acceptance are also critical. Clinicians must be trained not only in how to use the AI tool, but also in understanding its performance characteristics and limitations. For example, an AI risk model might be very good at predicting ER-positive cancers but less so triple-negative; if clinicians know this, they can contextualize risk scores for younger women who might develop aggressive subtypes. A survey of breast radiologists in Sweden found that while many were open to using AI, they wanted clarity on who is responsible if AI makes an error and guidance on how to incorporate AI results into decision-making [68,71]. Radiologists do not want to become complacent or blindly reliant on AI—they emphasize it should support, not replace, their expertise [31]. This calls for integration protocols where AI is consulted but the radiologist has final say, and discrepancies trigger a careful review rather than automatic override. Early experiences suggest that with time and familiarity, trust in AI can grow, especially if radiologists see that AI catches some cancers they might have missed or appropriately downgrades clearly benign cases [40].

On a program level, interdisciplinary coordination is needed. Implementing risk-based screening means engaging primary care (who often manage referrals to screening and high-risk counseling), genetic counselors (for integrating genomics), IT personnel, and administrative leadership [25,79,80]. For example, in the WISDOM trial, participants in the personalized arm receive communications explaining their risk and a tailored plan—the infrastructure for that includes call centers, web portals, and educational materials that standard programs did not need [12,63]. Integrating AI decisions also means.

Another consideration is interoperability and standards. Multiple vendors are producing AI tools, and not all will be used in a given center. Ensuring that AI risk scores or detection results from different sources can be integrated into one cohesive patient record or risk profile is a challenge. There are efforts to standardize how AI outputs (e.g., lesion probability scores) are encoded and communicated, akin to DICOM standards for images [80,81,82].

Finally, prospective clinical integration studies are needed: a lot of AI integration knowledge comes from either retrospective analyses or a few single-center trials.

## 9. Validation, Regulation, Clinical Evidence, and Ethical Implications

Before AI-driven personalization can be widely adopted, evidence must demonstrate safety and net benefit. To date, most approvals in breast imaging target detection/diagnosis; authorization for risk-based interval change or queue triage is emerging but remains limited [25,40,74,75,83,84]. Although this is rapidly evolving (as mentioned, an AI risk model (ProFound AI Risk) has gained FDA clearance [10]), regulators are cautious about any AI that could alter a standard care pathway (like extending intervals), since the downstream impact on cancer outcomes and population health must be considered [58].

Clinical validation for AI risk models ideally means prospective studies or randomized trials showing improved outcomes (or non-inferiority with reduced harms). For example, if implementing an AI personalized screening schedule, one would want to see that cancer detection is not inferior and that false positives and overdiagnoses are reduced—essentially a demonstration of net benefit.

The WISDOM and MyPeBS trials will provide some evidence here, though their risk algorithms are not purely AI-based (they incorporate conventional risk models with some genetic testing) [10,12,13,65]. It is conceivable that future trials will directly test an “AI-guided screening” strategy vs. standard. Until such data accrue, there is a reliance on modeling and retrospective studies. One such modeling study found that risk-stratified screening guided by an AI risk model could maintain cancer detection while reducing unnecessary screens and biopsies in low-risk groups, appearing cost-effective in certain scenarios [53]. Still, models are no substitute for real outcomes.

Regulators also focus on generalizability and bias. An AI might perform well in the dataset where it was developed but falter in a different healthcare setting or demographic group, highlighting the importance of evaluating AI on multiple external datasets and ensuring racial, ethnic, and age diversity in validation [3,76]. The study by Eriksson et al. [10] is a good model of extensive external validation (4 countries) before proposing clinical use: such rigorous validation builds confidence that an AI tool is robust and not overfitted to one context. Similarly, Omoleye et al. validated the MIRAI algorithms in an ethnically diverse population [21].

From a regulatory standpoint, once an AI is approved, using it in practice (especially to drive decisions like screening frequency) may require ongoing post-market surveillance [58,75]. In Europe, the Medical Device Regulation (MDR) has tightened requirements for AI tools, classifying many as high risk, which mandates continuous monitoring and periodic reassessment [75,85,86,87]. If an AI’s performance drifts over time (perhaps as cancer incidence or screening technology changes), there must be mechanisms to detect that. This is analogous to how human radiologists get re-credentialed or audited periodically; AI might require a form of re-validation or adaptation (some propose AI models should be allowed to update themselves with new local data, which raises further regulatory questions) [25].

One complication is that evaluation of screening interventions typically demands very large sample sizes and long follow-up (to see mortality impact, for example). Regulators might not require demonstrating mortality reduction for an AI tool (just as new screening modalities are often approved based on improved sensitivity rather than proven mortality benefit), but they will expect evidence of improved detection or reduced harm. For AI detection systems, enrichment reader studies and now prospective trials (like MASAI) provided evidence enough for use [43]. For AI risk models that direct screening, comparable evidence might be an RCT showing non-inferior detection with fewer false positives (which is essentially what WISDOM/MyPeBS are targeting without AI) [10,12,13,63]. If those trials are positive, even if the algorithm used is not the latest AI, it opens the door for substituting an AI-based risk tool that performs similarly or better. It will be incumbent on AI developers to demonstrate that their model’s risk stratification correlates with outcomes at least as well as the ones tested in trials.

Privacy is another concern, particularly as personalization often implies gathering more data (genetic tests, detailed personal info), and all these data need protection [25,58,74,88,89,90,91,92]. When AI algorithms are cloud-based or developed by third parties, strict data governance is required to ensure patient data is not misused. Deidentification and secure data pipelines can mitigate risks, but public sensitivity to health data breaches is high, so trust must be maintained.

There are also ethical questions around resource allocation and potential unintended consequences. If AI identifies a large high-risk group needing MRI, can the healthcare system accommodate that without siphoning resources from elsewhere? And what about those deemed low risk—will insurance pay for them to have less screening, and are we comfortable potentially missing some cancers in that group (since low risk is not zero-risk)? Society must grapple with the value judgments inherent in personalization: essentially stratifying who gets more or less medical intervention. Ideally, this is done to everyone’s benefit (reducing harm for low risk, improving detection for high risk), but if mismanaged, it could be seen as rationing care for some. Ethical implementation requires transparency about why changes are made (e.g., “Evidence shows this approach is better for your health on balance”) and ensuring that any savings from reduced interventions in low-risk groups are reinvested in improving care (for example, funding more comprehensive screening for high-risk groups or other preventive services) [25,58,88].

Notably, extending intervals for women labelled ‘low risk’ can increase false-negative exposure, particularly if model performance is weaker for ER-negative/TNBC or dense breasts. Programs should therefore define floor intervals (e.g., no extension below X months outside trials), recalibrate locally if subgroup drift is detected, and ensure that any ‘saved capacity’ funds earlier diagnostics for high-risk strata: these safeguards reduce the chance that personalization inadvertently widens disparities.

Finally, we must consider public perception and education. The success of personalized screening will depend on public acceptance. Misunderstandings could breed fear (“the computer is deciding my fate”) or unrealistic expectations (“the AI will catch everything, so I don’t need to be vigilant”) [26]. Engaging patient advocacy groups, communicating in plain language, and perhaps even allowing patients to see simplified outputs of the AI (like a risk level and explanation) can help demystify the process, as AI should augment the patient-provider relationship, not replace it. Ethically, maintaining respect and empathy in care is crucial; AI’s involvement should never make patients feel like “numbers” in an algorithm.

## 10. Conclusions

AI-driven personalization of breast cancer screening represents a paradigm shift in preventive oncology—moving from population-level heuristics to data-informed individualized strategies. Over the last decade, AI has accelerated the momentum toward individualized breast cancer screening, providing tools to address the long-standing dilemmas of population screening.

The transition from rigid age-based models to flexible, risk-based protocols is on the horizon, supported by a growing body of literature and early clinical experience. However, realizing this potential in routine care will require careful navigation of practical, regulatory, and ethical challenges.

A recurring theme of this review is balance: balancing innovation with validation, efficiency with equity, and automation with human touch. If we strike this balance, the outcome could be a smarter, more efficient screening paradigm that saves more lives while causing fewer harms. Such a paradigm—AI-driven yet patient-centric—would indeed fulfill the promise of precision medicine in cancer prevention, ensuring that each woman’s screening journey is as unique as her own risk, and as effective as current science allows.

Before altering intervals or modalities at scale, programs should confirm subgroup safety (particularly for dense breasts and ER-negative/TNBC phenotypes), encourage prospective studies for interval-cancer and stage-shift endpoints, and report outcomes by density/age/subtype; require local calibration and post-market surveillance with drift detection, institute equity audits to avoid widening disparities, adopt governance for model updates and human-in-the-loop oversight, and link economic evaluations to re-investment in high-risk care. Pending such evidence, interval extension for “low-risk” groups should be capped within monitored pathways.

## Figures and Tables

**Table 1:** Scope and terminology: four distinct AI functions in breast screening.

Risk prediction (future risk estimate at 1–5 years). Endpoint: calibration/discrimination; use: interval planning and modality selection.
Lesion detection/diagnosis (case-level/lesion-level cancer probability on current exam). Endpoint: sensitivity/specificity/recall.
Workflow triage (prioritization or auto-exclusion of likely negative exams). Endpoint: safety-audited workload reduction/time-to-diagnosis.
Clinical decision support (algorithm-informed schedules/modalities). Endpoint: net benefit on harms/benefits at program level.

**Table 2:** Conventional age-based vs. AI-driven screening models.

Aspect	Conventional Program	AI-Personalized Program	Representative Evidence
Basis for Eligibility	Broad age range (e.g., 50–69) regardless of individual risk.	Individual risk profile (genetics, breast density, prior images, etc.) used to stratify women.	Policies across 22 countries; ENVISION consensus; WISDOM/MyPeBS concepts.
Screening Interval and Modality	Fixed schedule (e.g., annual or biennial mammogram) for all. Supplemental MRI only for very high-risk (e.g., BRCA carriers) outside routine program.	Variable frequency and modality based on risk: high-risk women screened more often or with sensitive modalities (MRI); low-risk screened less frequently.	Optimization framework for risk-based intervals; MRI benefit in extremely dense breasts.
Cancer Detection	Proven mortality reduction, but ~20–25% of cancers occur between scheduled screens (interval cancers). One protocol for all can lead to missed cancers at higher risk and over-screening in low risk.	Improved early detection in simulations and trials by tailoring to risk. AI-driven policies achieved earlier detection than annual screening with 25% fewer mammograms. High-risk women can be identified for additional imaging, catching cancers that mammography misses.	Interval-cancer rates; ScreenTrustCAD and MASAI prospective data.
Harms and False Positives	High cumulative false-positive rate (~50% over 10 years) and notable overdiagnosis (~10–20%). Low-risk women undergo unnecessary imaging and biopsies.	Aimed at reducing harm by sparing low-risk individuals. Preliminary evidence shows similar or slightly lower recall rates when AI triage removes normal exams. Overdiagnosis may decline by screening fewer low-risk lesions, though long-term data are pending.	False-positive risk; overdiagnosis syntheses; prospective AI workload/recall findings.
Operational Efficiency	Resource-intensive (all exams read by radiologists; double reading in some programs) with many normal exams reviewed. Workforce shortages impact sustainability.	More efficient: AI triage can automate normal case dismissal and assist reads. Prospective trials show ~44% reduction in radiologist reading workload with AI support, with no loss in cancer detection. Potentially more cost-effective through targeted use of MRI and fewer overall screens.	Prospective trials show meaningful workload reduction with maintained detection.

**Table 3:** Subtype-specific and interval cancer findings reported for AI risk models (qualitative synthesis).

Studies/References	Model/Setting	Endpoint	Subtype/Interval-Cancer Signal	Notes
[16]	Mirai; multi-country validation	5-year risk (C-index/AUC)	Strong overall discrimination; limited published stratification by ER/TNBC; evidence gap acknowledged	Multi-institutional external validation
[22]	DL risk (UK screening)	3-year risk	Similar AUCs for screen-detected vs. interval cancers (~0.67–0.69); higher AUC for stage II+ (~0.72) implying signal for clinically significant disease	Subtype-specific ER/TNBC estimates not reported
[10,23,24]	Image-based short-term risk ± PRS	2-year risk	High-risk stratum enriched for larger/stage II tumors; fewer small ER+ in high-risk vs. low risk; generalizes across 4 EU cohorts	Supports targeted supplemental imaging; ER/TNBC-specific AUC gaps remain

**Table 4:** Domains and safeguards for integrating AI personalization into clinical screening programs.

Domain	Key Challenges	Practical Safeguards/Solutions
**Data Privacy and Governance**	- Protection of sensitive genomic and imaging data. - Potential misuse or breach of data privacy.	- Implementation of secure data systems (e.g., federated learning) - Transparent consent and governance frameworks.
**Algorithm Validation and Generalizability**	- IAI algorithms trained predominantly on limited demographic datasets. - Risk of biased or inaccurate predictions in diverse populations.	- Inclusion of diverse populations in training and validation. - Ongoing real-world performance monitoring and recalibration.
**Explainability and Transparency**	- AI models often perceived as “black boxes,” complicating clinician and patient trust. - Difficulties in informed decision-making.	- Development of explainable AI models with visual interpretability (e.g., heatmaps, feature attribution). - Clear communication strategies tailored to patient health literacy.
**Health System Readiness**	- Need for infrastructure to support risk assessments and supplemental imaging modalities. - Potential shortage of specialized resources (e.g., MRI availability).	- Incremental roll-out strategies starting with high-risk populations. - Investment in infrastructure and provider education.
**Equity and Access**	- Potential widening of disparities if advanced risk assessments are costly or limited to specific populations. - Risk of two-tier screening systems.	- Public or insurance coverage for risk assessments. - Policy measures ensuring equitable access and cost controls. - Community engagement and culturally tailored education.
**Regulatory Oversight and Liability**	- Lack of clear regulatory pathways for continuously evolving AI algorithms. - Ambiguity in legal accountability for AI-based clinical decisions.	- Development of adaptive regulatory frameworks by bodies (FDA, EMA). - Clear clinical guidelines defining roles and responsibilities of radiologists, institutions, and software providers.
